# Dietary n-3 PUFA May Attenuate Experimental Colitis

**DOI:** 10.1155/2018/8430614

**Published:** 2018-02-15

**Authors:** Cloé Charpentier, Ronald Chan, Emmeline Salameh, Khaly Mbodji, Aito Ueno, Moïse Coëffier, Charlène Guérin, Subrata Ghosh, Guillaume Savoye, Rachel Marion-Letellier

**Affiliations:** ^1^INSERM UMR 1073, UFR de Médecine-Pharmacie, 22 boulevard Gambetta, 76183 Rouen Cedex, France; ^2^Department of Gastroenterology, Rouen University Hospital, 1 rue de Germont, 76031 Rouen Cedex, France; ^3^University of Calgary, Gastrointestinal Research Group, Snyder Institute for Chronic Diseases, Calgary, AB, Canada; ^4^Center for Advanced IBD Research and Treatment, Kitasato Institute Hospital, Tokyo, Japan; ^5^Nutrition Unit, Rouen University Hospital, 1 rue de Germont, 76031 Rouen Cedex, France; ^6^Institute of Translational Medicine, University of Birmingham, Birmingham, UK

## Abstract

**Background:**

Inflammatory bowel diseases (IBD) occurred in genetically predisposed people exposed to environmental triggers. Diet has long been suspected to contribute to the development of IBD. Supplementation with n-3 polyunsaturated fatty acids (PUFA) protects against intestinal inflammation in rodent models while clinical trials showed no benefits. We hypothesized that intervention timing is crucial and dietary fatty acid pattern may influence intestinal environment to modify inflammation genesis. The aim of this study was to evaluate the dietary effect of PUFA composition on intestinal inflammation.

**Methods:**

Animals received diet varying in their PUFA composition for four weeks before TNBS-induced colitis. Colon inflammatory markers and gut barrier function parameters were assessed. Inflammatory pathway PCR arrays were determined.

**Results:**

n-3 diet significantly decreased colon iNOS, COX-2 expression, IL-6 production, and LTB4 production but tended to decrease colon TNF*α* production (*P* = 0.0617) compared to control diet. Tight junction protein (claudin-1, occludin) expressions and MUC2 and TFF3 mRNA levels were not different among groups. n-9 diet also decreased colon IL-6 production (*P* < 0.05).

**Conclusions:**

Dietary n-3 PUFA influence colitis development by attenuating inflammatory markers. Further research is required to better define dietary advice with a scientific rationale.

## 1. Introduction

Inflammatory bowel diseases (IBD) affects genetically predisposed people exposed to environmental triggers [1]. Amongst environmental factors, dietary habits have long been suspected to contribute to the development of IBD [2]. IBD patients often considered diet as a potential trigger for initiating the disease or causing a relapse [3], and this concept led to exclusion diets especially in children [4].

An increased incidence of IBD has been associated with diets high in animal protein. Indeed, association between dietary pattern (fat/protein) and Crohn's disease (CD) risk was found in a study from Japan [5] while increased consumption of animal protein has been associated with higher IBD risk in a study from France [6]. A systematic review demonstrated that this Western dietary pattern (high fat, high n-6 polyunsaturated fatty acids (PUFA), and high meat) is associated with an increased IBD risk [7]. More recently, a study was conducted in 103 IBD patients using food frequency questionnaire over 1 year and the authors found a positive association between meat intake and disease relapse [8]. Similarly, Western diet had a deleterious impact on gut barrier function and dysbiosis in IBD murine models [9].

While n-3 and n-6 PUFA are essential in human nutrition, a Western diet is characterized by an unbalanced ratio of both types of PUFA (n-3/n-6 ratio). Indeed, linoleic acid (LA, n-6 PUFA) consumption has markedly increased (3-fold throughout the 20th century) [10]. Numerous epidemiological studies highlighted the role of dietary intake of monounsaturated fatty acids (MUFA) or PUFA in ulcerative colitis (UC) development. Higher intake of LA is associated with an increased risk of UC [11], while docosahexaenoic acid (DHA) (n-3 PUFA) [12] or oleic acid (n-9 MUFA) [13, 14] consumption is beneficial. Ananthakrishnan et al. found that greater fish intake was associated with lower risk of CD [15].

We and others demonstrated an anti-inflammatory effect of n-3 polyunsaturated fatty acids in rodent IBD models [16–21] while clinical trials failed [22]. We hypothesized that intervention timing is crucial and dietary fatty acid pattern may influence intestinal environment to modify inflammation genesis [23].

The aim of the study was to investigate the dietary influence of fatty acid composition before the onset of intestinal inflammation by administration of 2,4,6-trinitrobenzene sulfonic acid (TNBS). For this purpose, rats were fed with diets varying in n-3/n-6/n-9 ratio to reproduce dietary pattern from a pragmatic to a Western diet.

## 2. Materials and Methods

### 2.1. Animals and Study Design

**Figure 1:**
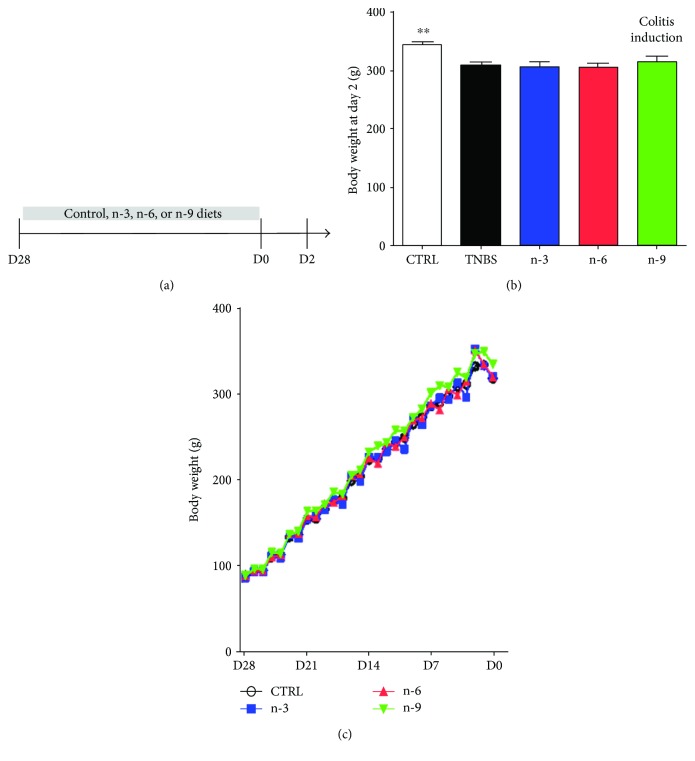
Experimental design and clinical parameters in rats receiving diets varying in their unsaturated fatty acid composition followed by TNBS-induced colitis. (a) Experimental design. Rats received diets varying in their PUFA composition for four weeks before colitis induction at day 0. Rats were killed at day 2. (b) Body weight at day 2. (c) Body weight follow-up from day 28 to day 2. ∗∗ means *P* < 0.01 versus all colitis groups (TNBS, n-3, n-6, and n-9).

Young Sprague-Dawley male rats weighing 75–100 g were purchased from Janvier (Le Genest St. Isle, France) and allowed to access food and water ad libitum. After 1 week acclimatization, 50 rats were randomly divided into 5 experimental groups; the control (CTRL) group was fed with control diet and received the vehicle, while colitic groups including TNBS, n-3, n-6, and n-9 groups were fed with control diet, n-3 diet, n-6 diet, and n-9 diet, respectively, and received TNBS for the colitis induction. Weight changes throughout the study were monitored every day. After 4 weeks of experimental diets (day 28 to day 1), the rats underwent 24 hours food deprivation prior to the TNBS or vehicle administration. During the colitis induction (day 0 to 2), rats were provided control diet. The overview of experimental design is illustrated in Figure 1(a).

All animal handling and treatment procedures were performed in accordance with both French national regulations and European Union regulations (Official Journal of the European Community L 358, 18/12/1986) and RML is authorized to use this animal protocol by the French government (Authorization n°76-106).

### 2.2. Diets

Four types of isocaloric and isolipidic experimental diets were prepared with several fatty acid proportions:

- The normal diet matched a balanced diet with a n-3/n-6/n-9 ratio equal to 1 : 4 : 16 as a fat ratio recommended by dietary guidelines and described as a well-balanced diet in the literature [24]. The control diet was given to CTRL and TNBS groups. The recommended dietary n-6/n-3 ratio is about 4 in human nutrition which is comparable to the control diet in this study.
- n-3 diet had a n-3/n-6/n-9 ratio equal to 1 : 1 : 4. We chose a n-3/n-6 ratio equal to 1 : 1 because this ratio was a target by dietary advice in a Japanese clinical trial for IBD patients [25]. In addition, a n-3/n-6 ratio is commonly used in experimental studies investigating the effect of n-3 therapy [24].
- n-6 diet fitted the Western diet with a n-3/n-6/n-9 ratio at 1 : 16 : 16. The dietary n-6/n-3 ratio is about 15 in human Western diets [26], and this ratio is useful to underline the imbalance that characterizes Western diets.
- n-9 diet had a similar n-3/n-6 ratio to CTRL diet but is enriched in n-9 MUFA. This n-3/n-9 ratio equal to 1 : 24 is comparable to the ratio observed in people following the Mediterranean diet [26].

Detailed diet composition is shown in Table 1.

### 2.3. Induction of Colitis

Administration of TNBS (Sigma-Aldrich Company, Saint-Quentin-Fallavier, France) was employed for colitis induction as previously described [16] in TNBS, n-3, n-6, and n-9 groups (colitic groups). The rats were sacrificed using anesthetic reagents (ketamine and xylazine) at day 2 for further analyses.

### 2.4. Western Blot

PBS, protease inhibitor cocktail, and phosphatase inhibitor cocktail were purchased from Sigma-Aldrich (Saint-Quentin-Fallavier, France). The 4–12% NuPAGE gels and SeeBlue multicolored standard were obtained from Invitrogen (Cergy-Pontoise, France). Frozen colon samples were homogenized in PBS with 0.1% protease inhibitor cocktail and 1% phosphatase inhibitor cocktail. Homogenates were centrifuged (12,000*g*, 15 min, 4°C) and the supernatants were collected. Protein concentration was determined following Bradford's colorimetric method. Aliquots of supernatants containing equal amounts of protein (30 *μ*g) were separated on 4–12% NuPAGE and then transferred to a nitrocellulose membrane (Hybond, GE Healthcare, UK). The mouse monoclonal antibody anti-PPAR*γ* (sc-7273), the goat polyclonal anti-COX-2 (sc-1747), the mouse anti-iNOS (sc-7271), the rabbit polyclonal anti-HNF-4 (sc-8987), and the HRP-conjugated secondary antibodies were obtained from Santa Cruz Biotechnology (Tebu, Le Perray-en-Yvelines, France). The rabbit anti-claudin-1 and the mouse anti-occludin were, respectively, obtained from Life technology and Invitrogen. After blocking, membranes were incubated with specific primary antibodies at the dilution of 1 : 100 (iNOS), 1 : 500 (COX-2, HNF-4, and PPAR*γ*), and 1 : 1000 (claudin-1, occludin. After three washes, membranes were then incubated with the secondary HRP-linked anti-goat IgG (for COX-2), anti-rabbit IgG (for HNF-4, claudin-1), and anti-mouse IgG (for iNOS, PPAR*γ*, and occludin) antibodies. The enhanced chemiluminescence light-detecting kit (GE Healthcare, USA) was utilized for immunodetection. Densitometric data were measured following normalization to the housekeeping protein (*β*-actin) by a Scientific Imaging Systems (Image QuantTL, GE Healthcare).

### 2.5. RNA Isolation and Gene Expression Analyses

Colon samples were frozen in liquid nitrogen and stored at −80°C before RNA preparation. Total RNA was isolated from rat colon specimens using a commercial RNA purification kit (SV total RNA isolation kit, Promega, Madison, WI) and mRNA expression of Muc2 (primer sequences F: CCTTGCTCTGCCATACCCGT, R: ACACTGGTCCTCTCCTCCCT) and TFF-3 (F: TAACCCTGCTGCTGGTCCTG, R: GTTTGAAGCACCAGGGCACA), and the internal control (GAPDH) was measured by qRT-PCR. Furthermore, gene expressions in Toll-like receptor signaling pathway were determined by real-time PCR array according to the manufacturer's protocol (PAMM-0018ZD, SA Biosciences, Frederick, MD) on CFX96 thermocycler (Bio-Rad, Hercules, CA). Data are expressed in fold regulation. The fold change (fold difference) is calculated by the equation 2(−∆∆CT). For the fold regulation, the software transforms fold change values less than 1 (meaning that the gene is downregulated) by returning the negative inverse.

### 2.6. Colon Cytokines and LTB4 Production

Concentrations of TNF*α*, IL-1*β*, and LTB4 in the colon homogenates were detected by ELISA (R&D Systems, Lille, France) following the manufacturer's instructions.

### 2.7. Proteolytic Pathway Activities

The evaluation of proteolytic activities (caspase-like and chymotrypsin-like) was performed by spectrofluorometric microtiter plate fluorometer (Mithras LB 940, Berthold Technologies) using fluorogenic proteasome substrate in the presence or absence of specific proteasome inhibitors as previously described [27].

### 2.8. Statistical Analysis

Statistical comparisons were performed using GraphPadPrism 5. Data are expressed as mean ± SEM. Body weight changes and food intake were analyzed with 2-way ANOVA for repeated measures with Tukey's post hoc tests. All the other variables were analyzed by one-way ANOVA with Bonferroni post hoc test or Kruskal-Wallis test as appropriate. Differences were considered significant at *P* < 0.05.

## 3. Results

### 3.1. TNBS-Induced Colitis Decreased Body Weight

Colitic groups had a lower body weight compared to control rats (*P* < 0.01) at day 2 but there is no difference among colitic groups (Figure 1(b)).

### 3.2. TNBS-Induced Colitis Increased Inflammatory Markers

**Figure 2:**
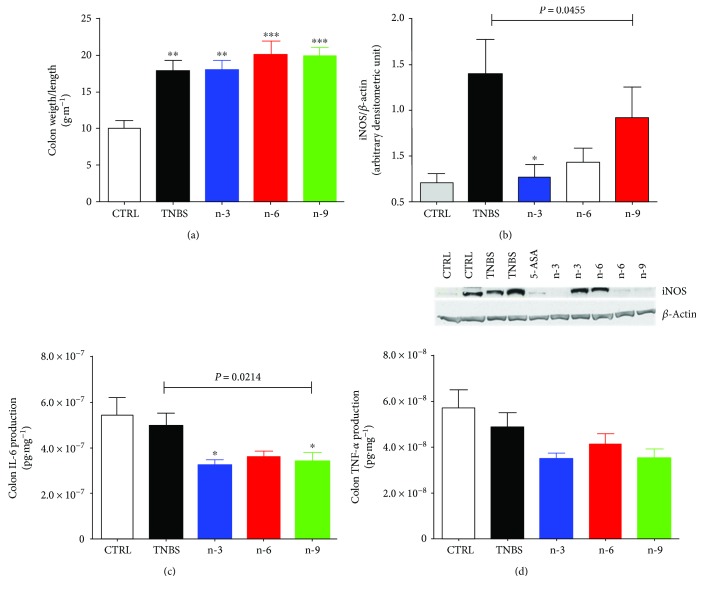
Inflammatory markers in rats receiving diets varying in their unsaturated fatty acid composition for 4 weeks followed by TNBS-induced colitis. (a) Colon/weight length at day 2. (b) Colon iNOS expression with a representative gel at day 2. (c) Colon IL-6 and (d) TNF*α* production. 5-Aminosalicylic acid (5-ASA) is used a positive anti-inflammatory control. Data from colitic rats were compared by 1-way ANOVA followed by Tukey posttests. ∗∗ means *P* < 0.01 versus CTRL, ∗∗∗ means *P* < 0.001 versus CTRL, and ∗ means *P* < 0.05 versus TNBS.

Colon weight/length ratio was increased in colitic rats compared to control group (*P* < 0.01 for TNBS and n-3, *P* < 0.001 for n-6 and n-9, Figure 2(a)) without significant differences among colitic groups (Figure 2(a)). Colon iNOS was significantly higher in colitic groups compared to control group (*P* = 0.0141, Figure 2(b)).

### 3.3. n-3 Diet Decreased Colon Inflammatory Markers

Among colitis groups, n-3 group had a lower colon iNOS compared to TNBS group (*P* < 0.05, Figure 2(b)). Colon IL-6 production was significantly lower in n-3 and n-6 groups compared to TNBS group (*P* < 0.05 for both, Figure 2(c)) while colon TNF*α* production did not significantly differ among colitis groups (Figure 2(d)) but tend to decrease in n-3 group compared to TNBS group (*P* = 0.0617). Transcription factors HNF-4*α* and PPAR*γ* expressions were not different among groups (data not shown).

### 3.4. n-3 Diet Decreased COX-2 Expression and LTB4 Production in the Colon

**Figure 3:**
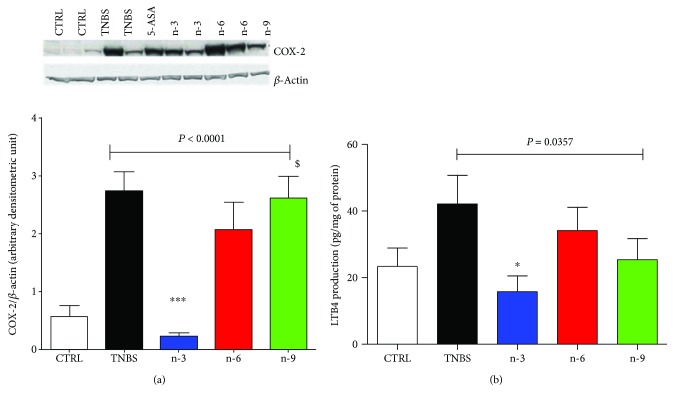
Eicosanoid pathway in rats varying in their unsaturated fatty acid composition for 4 weeks followed by TNBS-induced colitis. (a) Colon cyclooxygenase-2 (COX-2) expression with a representative gel and (b) colon LTB4 production at day 2. 5-Aminosalicylic acid (5-ASA) is used a positive anti-inflammatory control. Data from colitic rats were compared by 1-way ANOVA followed by Tukey posttests. ∗ means *P* < 0.05 versus TNBS, ∗∗∗ means *P* < 0.001 versus TNBS, and $ means *P* < 0.05 versus n-3.

Among colitis groups, n-3 group had a lower colon COX-2 expression compared to TNBS group (*P* < 0.001, Figure 3(a)). In addition, colon LTB4 production was lower in the n-3 group compared to TNBS group (*P* < 0.05, Figure 3(b)).

#### 3.4.1. Gut Barrier Function Was Not Affected by Dietary Treatments

**Figure 4:**
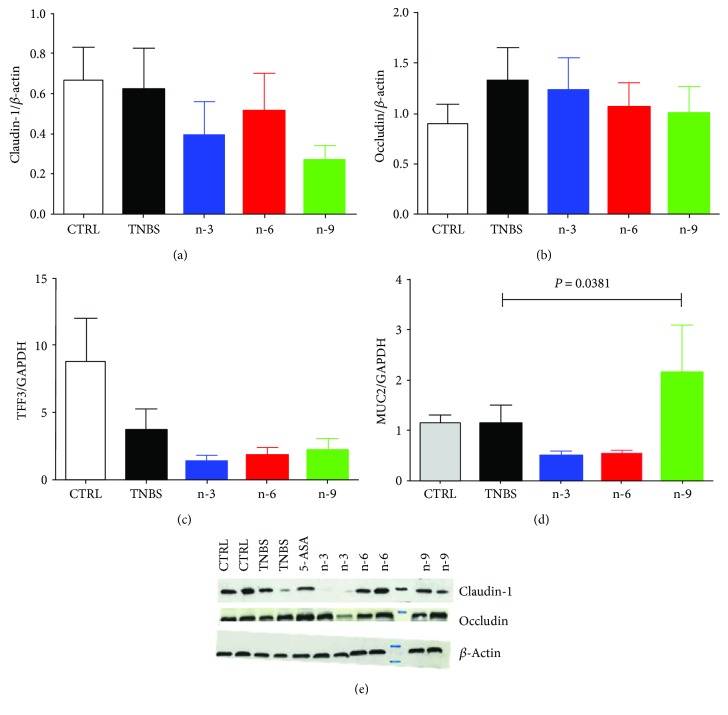
Gut barrier parameters in rats receiving diets varying in their unsaturated fatty acid composition for 4 weeks followed by TNBS-induced colitis. (a) Colon claudin-1 expression, (b) occludin expression, (c) trefoil factor 3 (TFF3) mRNA level, and (d) MUC2 mRNA level at day 2. (e) Representative gel of colon claudin-1 and occludin expression. 5-Aminosalicylic acid (5-ASA) is used a positive anti-inflammatory control. Data from colitic rats were compared by 1-way ANOVA followed by Tukey posttests.

Tight junction proteins claudin-1 and occludin were not different among groups (*P* = 0.4750 and *P* = 0.8553, resp., Figures 4(a) and 4(b)). TFF3 mRNA levels were not different among groups (*P* = 0.3729, Figure 4(c)). MUC2 mRNA levels were not different among groups (1-way ANOVA, *P* = 0.0381, posttests *P* > 0.05, Figure 4).

### 3.5. Colitis or Dietary PUFA Did Not Modify Proteasome Activity

Chymotrypsin and trypsin-like activities were not different among colitic groups (*P* = 0.3510 and *P* = 0.0651, resp., data not shown).

### 3.6. Dietary Modulation of Inflammatory Gene Expression

In colitis groups, n-3 diet upregulated IL-1A, TLR-2, and MA2K3 genes while n-9 diet upregulated TLR-4 genes (*P* = 0.044, *P* = 0.013, and *P* = 0.021, resp., Table 2). n-6 upregulated HMGB1 (*P* = 0.042) without affecting TLR pathways (*P* > 0.05, Table 2).

## 4. Discussion

Numerous experimental studies found anti-inflammatory effects of n-3 PUFA in intestinal inflammation while randomized clinical trials failed to demonstrate efficacy [2, 23]. We previously hypothesized that the discrepancy between clinical trials and experimental studies could result from the timing of the intervention [23]. In our previous studies in colitis models [16–19], we tested nutritional intervention with n-3 PUFA in a curative manner. We now speculated that nutritional intervention with fatty acid should be preventive as reflected in the epidemiological studies. In epidemiological studies, dietary intake of PUFA modifies IBD risk, and identification of their potential mechanisms is now required. To this purpose, we fed rats for four weeks with diets differing in their PUFA composition before the onset of colitis.

In the present study, n-3 diet downregulated colon iNOS expression (Figure 2(a)) in rats with TNBS-induced colitis similar to previous studies performed by us [16, 19] or others [21]. Indeed, n-3 PUFA can regulate oxidative stress. Camuesco et al. found that olive oil enriched with fish oil decreased oxidative activity by restoring glutathione concentration and reducing iNOS expression in the colon of rats [21]. Dietary n-3 PUFA exerted anti-inflammatory properties. Indeed, n-3 diet decreased colon COX-2 and colon LTB4 production (Figure 3). This result is in accordance with our previous studies showing an inhibitory effect of nutritional intervention with n-3 PUFA on COX-2 and LTB4 [16, 19]. Similarly, it has been shown that antagonizing arachidonic acid-derived eicosanoids reduced inflammation and colitis severity in mice [28]. In addition, alteration of eicosanoids is one of the PUFA main mechanisms [29]. In the present study, n-3 diet also downregulated colon proinflammatory cytokines such as IL-6 (Figure 2(c)) and tend to decrease TNF*α* production.

In the present study, IL-1A gene expression was upregulated by n-3 diet (Table 2). This result is in accordance with an *in vitro* study showing that EPA treatment increased IL-1A secretion in human keratinocytes [30]. In our study, we observed a significant decreased IL-6 production (Figure 2(c)) while IL-6 gene expression did not differ (Table 2). The discrepancy between gene expression and protein concentration is a frequent finding in the literature. In a previous study, we observed that TNBS administration led to a 60% increase of TNF*α* production, while a 12-fold increase of gene expression was observed [16]. Studies that have tested correlations between gene expression and protein levels have found that mRNA and protein abundances are differentially expressed, suggesting a frequent posttranscriptional regulation of gene expression [31].

Dietary n-3 PUFA increased TLR2 gene compared to control diet while n-9 diet increased TLR4 gene (Table 2). In the literature, the inhibitory effect of n-3 PUFA on TLR2 is controversial. TLR2 protein expression was downregulated by EPA in mouse adipose stem cells [32] while a study investigating the effect of a range of saturated and unsaturated fats on TLR2 and TLR4 activation found no effect [33]. The investigators of this study did not find any effect on DHA, EPA, or oleic acid to activate TLR2 and TLR4 in HEK-Blue cells [33]. Nevertheless, these fatty acids were able to downregulate cytokine production such as TNF*α*, IL-6, and MCP-1 secretion in human adipose tissue and adipocyte cultures [33]. We studied dietary effects on TLR expression but we did not explore their effects on the intestinal microbiota. It has been demonstrated that fish oil is able to attenuate n-6 PUFA-induced dysbiosis in a colitis model [34].

Dietary n-6 increased gene expression of high-mobility group box 1 (HMGB1, Table 2). An increased of colon HMGB1 by dietary n-6 PUFA was observed in rats with colon cancer [35]. HMGB1 can activate multiple signaling pathways such as TLR but we did not observe any increase in TLR signaling by n-6 diet. Other signaling pathways such as receptor for advanced glycation end products (RAGE) signaling may be involved [36]. Indeed, increased RAGE via dietary n-6 has been reported in experimental colon cancer models [37].

Except for il-1a, MAP2k3, and TLR genes, we observed only modest effect of n-3 diet in inflammatory gene expression. Contrary to other studies, we aimed to evaluate a dietary effect on n-3 PUFA before inflammation genesis while numerous studies are interested in a pharmacological effect of n-3 PUFA in a curative manner [21, 38]. Numerous studies have used long chain n-3 PUFA [39] while the experimental diets used in the present study did not contain any long chain PUFA; these diets cannot directly reproduce a typical omnivore human diet. Route of administration is also a crucial point, and we used diets varying in their unsaturated fatty acid composition while n-3 PUFA are often administered by gavage. These experimental design discrepancies may explain our effects on inflammatory gene expression.

Fatty acids are endogenous ligands for HNF-4*α* [40], and the role of HNF-4*α* in the intestinal inflammatory homeostasis has been demonstrated in mice with the intestinal epithelial deleted HNF-4*α* [41]. We hypothesized that dietary PUFA can regulate HNF-4 but we did not observe any modifications of colon HNF-4*α* expression among groups. Similarly, nuclear receptor PPAR*γ* can be activated by PUFA and is a regulator of intestinal inflammation [42, 43], but its expression is not different among groups.

Dietary PUFA did not affect barrier function in our study. We investigated tight junction proteins, MUC2 and TFF3 mRNA levels, and we did not find any significant effect among groups (Figure 4). Some studies found a protective effect of n-3 PUFA on barrier function. Hudert et al. have used transgenic mice carrying the *C. elegans fat-1* gene encoding an n-3 fatty acid desaturase that converts n-6 to n-3 fatty acids and they induced DSS colitis in these mice [44]. They found that *fat-1* mice were protected from colitis induction compared to wild-type mice with decreased inflammatory markers [44]. They also found that fat-1 mice exhibited an increased production of protective markers such as TFF3 [44]. Fish oil supplementation in rats with TNBS-induced colitis also increased the number of goblet cell with mature mucin granules [38]. Nevertheless, our experimental design is different from these studies. Indeed, we investigated the effect of PUFA at a dietary dose while the previous studies investigated PUFA as immunonutrients.

In the present study, n-3 diet group which showed n-3/n-6 ratio equals to 1 attenuated inflammatory markers in the colon. This preventive approach has been already tested in small clinical trials. In a Japanese study, the efficacy of n-3 diet therapy in IBD patients has been already evaluated [25]. The authors of this study combined a double nutritional approach to achieve a n-3/n-6 ratio of 1 for their patients by dietary advice and nutritional supplementation [25]. Their patients were prohibited from consuming the main source of n-6 PUFA consumption such as vegetable oils or dressings. They also provided a n-3 PUFA food exchange table to privilege and n-3 supplementation [25]. The authors of this study found a higher n-3/n-6 ratio in the remission group [25]. In a Norwegian study, they evaluated the effect of 600 g of salmon consumption per week for 8 weeks in 12 active UC patients and they found decreased clinical inflammatory index [45]. A proof of concept study is now required to evaluate n-3 PUFA in a preventive manner. As we cannot directly target IBD physiopathology with a nutritional therapy before the IBD diagnosis, we may first evaluate n-3 therapy in CD postoperative patients. Indeed, postoperative phase is considered as a perfect window to evaluate predisposing factors to IBD recurrence.

Similarly, in a recent epidemiological study, women with a prudent diet (characterized by greater intake of fruits, vegetables, and fish) had a lower CD risk [15]. In addition, greater intake of fish (*P* trend = 0.01) has been specifically associated with lower risk of CD [15].

In conclusion, prudent diet with a high n-3/n-6 ratio may contribute to partially limit colitis genesis. Further research will be mandatory to determine mechanisms underlying dietary effects to better define dietetic advice with a scientific rationale.

## Figures and Tables

**Table 1:** Fatty acid composition of the experimental diets.

	CTRL	n-3 diet	n-6 diet	n-9 diet
Total fat (g/1000 g of diet)	49.7	49.4	49.7	49.8
Saturated fat (g)	10.2	9.9	9.3	9.6
MUFA (g)	29.8	26.2	20.1	32.8
PUFA (g)	9.8	13.3	20.3	7.4
n-6 fatty acids (g)	7.9	7.3	19.1	6.1
n-3 fatty acids (g)	1.8	6.1	1.2	1.3
n-9 fatty acids (g)	29.3	25.8	19.8	32.2
n-3/n-6/n-9 ratio	1 : 4:16	1:1 : 4	1 : 16:16	1:4:24

**Table 2:** Inflammation pathway PCR array in rats receiving diets varying in their unsaturated fatty acid composition followed by TNBS-induced colitis. Colon RNA from rats receiving diets varying in their unsaturated fatty acid composition before the onset of TNBS-induced colitis. The results were compared to rats receiving TNBS with a control diet. Data in bold are significantly different from TNBS rats.

	n-3 diet		n-6 diet		n-9 diet	
	Fold regulation	*P* value	Fold regulation	*P* value	Fold regulation	*P* value
Il1a	**2.29**	**0.044**	1.33	0.224	1.50	0.157
Il1b	1.60	0.125	1.09	0.776	−1.03	0.879
Il12a	−1.64	0.113	−1.12	0.532	−1.41	0.226
Il6	1.54	0.363	−2.76	0.219	−1.10	0.656
TNF	1.40	0.138	1.32	0.260	1.24	0.393
Ifng	1.06	0.417	2.30	0.124	1.78	0.183
Il10	1.49	0.378	−1.36	0.793	1.16	0.776
Il1r1	1.39	0.205	1.26	0.446	1.11	0.864
Hmgb1	1.09	0.492	**1.52**	**0.042**	1.02	0.851
Map2k3	**1.65**	**0.021**	1.36	0.117	1.2	0.278
Il2	−1.27	0.904	4.23	0.258	3.13	0.275
Clec4e	1.46	0.372	−2.13	0.184	−1.37	0.724
Lta	−1.04	0.572	−2.44	0.351	−1.09	0.952
Cd86	−1.11	0.569	−1.12	0.778	−1.52	0.163
Fos	−1.05	0.345	−1.3	0.379	−1.32	0.214
Irf1	−1.32	0.451	1.08	0.988	1.02	0.693
Jun	−1.37	0.340	1.09	0.762	−1.34	0.158
Tlr1	1.23	0.419	−1.05	0.803	1.06	0.654
Tlr2	**1.73**	**0.013**	1.18	0.320	1.16	0.371
Tlr3	−1.38	0.320	1.25	0.659	1.11	0.846
Tlr4	1.49	0.140	1.34	0.251	**2.01**	**0.005**
Tlr5	−1.09	0.805	1.18	0.815	1.49	0.758
Tlr6	1.26	0.381	1.02	0.855	1.05	0.960
Tlr7	−1.37	0.397	−1.44	0.207	−1.29	0.299
Tlr9	−1.24	0.802	−1.18	0.877	−1.47	0.285
